# LncRNA NCK1-AS1 promotes proliferation and induces cell cycle progression by crosstalk NCK1-AS1/miR-6857/CDK1 pathway

**DOI:** 10.1038/s41419-017-0249-3

**Published:** 2018-02-07

**Authors:** Haiyu Li, Yongqin Jia, Junning Cheng, Geli Liu, Fangzhou Song

**Affiliations:** 1 Molecular Medicine and Cancer Research Center, Chongqing Medical University, Chongqing, 400016 China; 2 Department of Biochemistry and Molecular Biology, Chongqing Medical University, Chongqing, 400016 China; 3 The Second Clinical College of Chongqing Medical University, Chongqing Medical University, Chongqing, 400016 China

## Abstract

The purpose of this study was to develop an lncRNA signature to improve the prediction of the prognosis of cervical cancer through integration bioinformatics and analysis of TCGA RNA sequencing data. In this study, we established a set of four lncRNA signatures that was significantly associated with recurrence-free survival using the Cox regression model. Functionally, we screened the CC-associated lncRNA NCK1-AS1 as a new candidate lncRNA and regulator which promotes development and progression in CC. qRT-PCR and RNA in situ hybridization (RISH) results showed that NCK1-AS1 was significantly up-regulated in 77.4% (24/31) of the CC tissue group compared with the normal group (*P* < 0.01). Interestingly, we demonstrated that transcription factor SP1 directly binds to the promoter to activate NCK1-AS1 expression in SiHa cells. In vitro and in vivo assays of silencing NCK1-AS1 significantly inhibited cell proliferation and invasion, with induction of cell arrest in S phase of the cell cycle. Furthermore, Human Transcriptome Array 2.0 analysis after NCK1-AS1 silencing highlighted alterations in cell proliferation and cell cycle pathways. NCK1-AS1 functioned as a molecular sponge for miR-6857, antagonizing its ability to repress CDK1/6 protein translation. In conclusion, these findings suggest that NCK1-AS1/miR-6857/CDK1 crosstalk serve as a critical effector in cervical cancer progression and may serve as a potential target in cervical cancer.

## Introduction

Cervical cancer is the fourth-most common cause of cancer and deaths of cancer in women worldwide^1–3^. Despite the great efforts that have been made on HPV vaccines to protect women from cervical cancer, this malignancy remains the second-most common cause of female-specific cancer after breast cancer^4^. Nearly 80% of cervical cancers occur in developing countries. Squamous cell cancer is the cervical cancer with the greatest incidence. Unfortunately, there are no definite diagnostic and prognostic biomarkers^5,6^.

Increasing evidence demonstrates that long non-coding RNA (lncRNA) expression is tissue-specific and frequently dysregulated in various types of cancers,and some lncRNAs are correlated with cancer recurrence and poor prognosis^7–9^. LncRNAs are defined as non-protein coding transcripts longer than 200 nucleotides, and were initially thought to represent spurious transcriptional noise. In general, the majority (~78%) of lncRNAs are characterized as tissue-specific, as opposed by only ~19% of mRNAs. In addition to higher tissue specificity^8,10–12^, lncRNAs are characterized by higher developmental stage specificity, and cell subtype specificity in heterogeneous tissues, such as human neocortex^13,14^. Large-scale sequencing of cDNA libraries and more recently transcriptomic sequencing by next generation sequencing indicate that long noncoding RNAs number are in the order of tens of thousands in mammals^15–17^. However, despite accumulating evidence suggesting that the majority of these are likely to be functional, only a relatively small proportion has been demonstrated to be biologically relevant. HOTAIR originates from the HOXC, which represses transcription across 40 kb of the HOXD locus by altering chromatin trimethylation state. To achieve this, HOTAIR directs the action of Polycomb chromatin remodeling complexes in trans to govern the cells’ epigenetic state and subsequent gene expression^18–20^. Components of the Polycomb complex^21^, including Suz12, EZH2, and EED, contain RNA binding domains that may potentially bind HOTAIR and probably other similar ncRNA^22–25^. Recent evidence has raised the possibility that transcription of genes that escape from X-inactivation might be mediated by expression of lncRNA within the escaping chromosomal domains^26,27^. The ability of lncRNAs regulating associated protein-coding genes may contribute to disease if mis-expression of an lncRNA deregulates a protein coding gene with clinical significance. In a similar manner, an antisense lncRNA that regulates the expression of the sense BACE1 gene, a crucial enzyme in Alzheimer’s disease etiology, exhibits elevated expression in several regions of the brain in individuals with Alzheimer’s disease^28,29^.

Previous studies provide several that lncRNAs are involved in CC progression, such as lncRNA-EBIC, TI10124, TI18382, TI21327, TI18318, TI22687, TI09485, and ASK00420. LncRNA-EBIC was founded to be an oncogenic lncRNA, which could promote tumor cell invasion in CC by binding to EZH2 and inhibiting E-cadherin expression. There are still exists a large number of previously unexplored lncRNA alterations in CC, especially in the expression patterns of CC-specific lncRNAs^30^. To investigate aberrantly expressed lncRNAs in cervical cancer, we integrated bioinformatics and analysis of TCGA RNA sequencing data (13 normals and 306 cervical squamous cell carcinomas and endocervical adenocarcinomas). Using the Cox regression model, we identified a prognostic four-lncRNA signature from TCGA data. In this comprehensive characterization of aberrantly expressed lncRNAs, we identified a CC specific upregulated lncRNA named as NCK1-AS1, which is a new candidate lncRNA promoting development of CC; and its biological role and molecular mechanism were evaluated as well.

## Results

### Identification of CC-specific and prognostic lncRNAs from TCGA CESC data

**Fig. 1:**
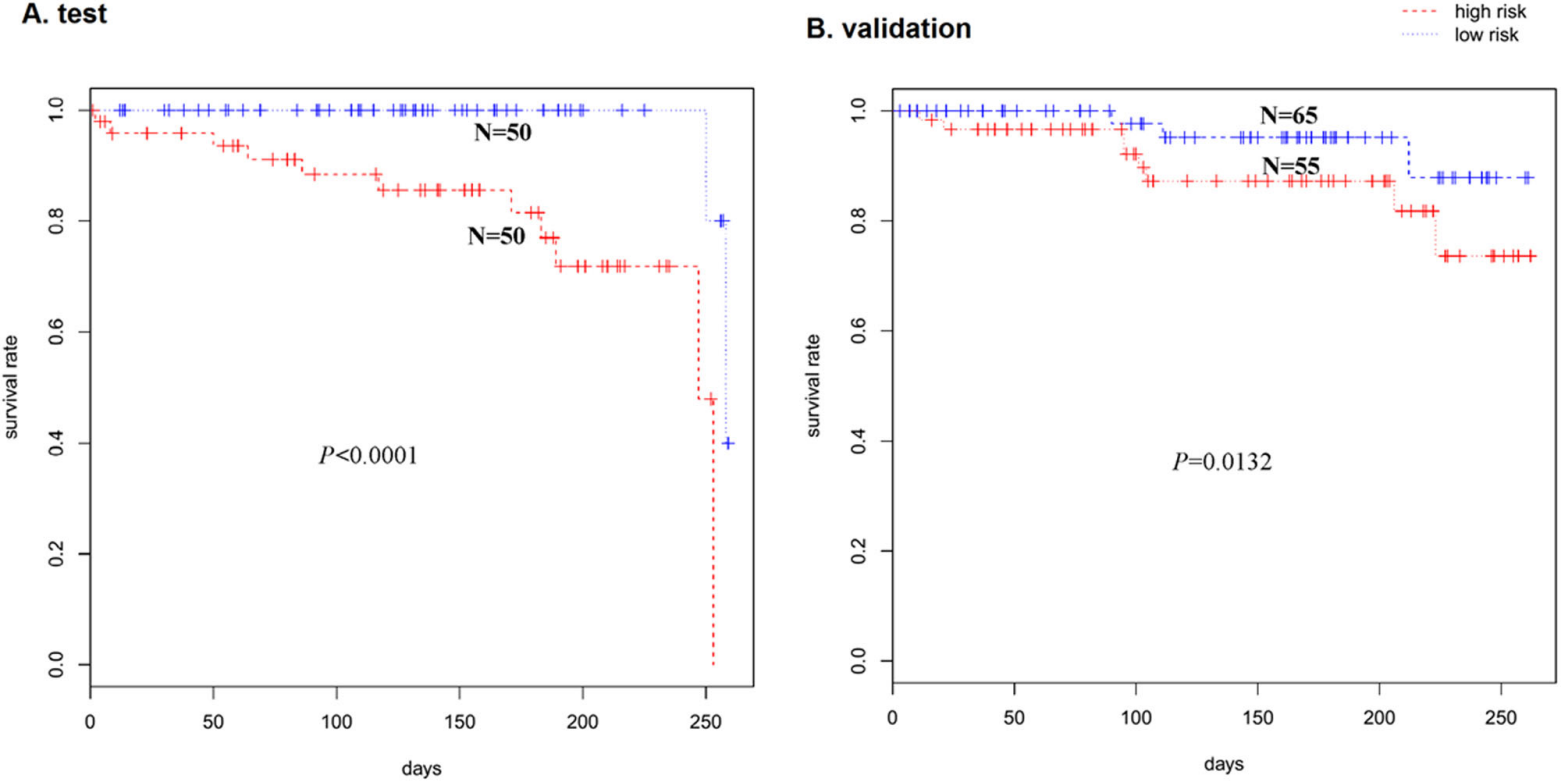
Kaplan–Meier curve of the cervical cancer survival from TCGA patients using the four-lncRNA risk-score formula. **a** Kaplan–Meier curves for test series (*N* = 100); **b** Kaplan–Meier curves for validation series (*N* = 120)

To detect prognostic lncRNAs that involved in cervical cancer progression, we subjected analysis of TCGA cervical squamous cell carcinoma and endocervical adenocarcinoma RNA sequencing data by univariable Cox proportional hazards regression analysis. A set of four-lncRNA signatures were significantly correlated with patients’ survival (*P* < 0.0001; Table 1). Based on the expression of these four lncRNAs for DFS prediction, established a risk-score formula as follows: Risk score = (0.7521141×expression level of AATBC) + (1.0531199×expression level of NCK1-AS1) + (1.0104329×expression level of LINC00937) + (−0.4792615×expression level of LINC00173) Then, the four-lncRNA expression signature risk score for each patient in the training set were calculated. Patients were divided into low-risk (*n* = 50) and high-risk (*n* = 50) groups according to the median risk score as cut-off in the training set. As shown in Fig. 1a patients in the high-risk group had significantly shorter survival times than those in the low-risk group (*P* < 0.0001). To confirm the risk-score formula, the risk scores for another 120 patients in testing sets were calculated. Consistent with the above-described result, patients in the high-risk group had significantly shorter survival times than those in the low-risk group (*P* = 0.0132) (Fig. 1b).

**Table 1:** Four lncRNAs were significantly associated with the CC survival in the test data set

Gnene symbole	Coefficient	Hazard ratio	*P* value cox	*P* value permutation
AATBC	0.7521141	0.471369	6.69E-04	0.0004
NCK1-AS1	1.0531199	2.8665807	3.25E-05	0.00234
LINC00937	−1.0104329	0.3640613	3.06E-05	0
LINC00173	−0.4792615	0.6192405	1.03E-02	0.0064

### GO and pathway analysis of four-lncRNA signature associated biological processes in CC

**Fig. 2:**
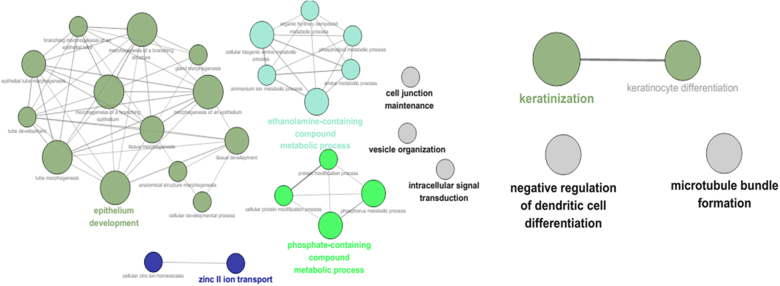
GO of three prognostic lncRNAs in test series. Each node in the graph represents a GO BP term. The color of the items separates them by similar function. The smaller *p* value of the term, the greater the node

Gene Ontology (GO) functional enrichment analysis was performed to identify associated biological processes and signaling pathways according to the risk score for classification. These results were visualized as interaction networks with Cytoscape. Four-lncRNA were mainly enriched in GO terms related to biological processes (BP) such as epithenlium development, intracellular signal transduction, cell junction maintenance microtubule bundle formation, negative regulation of dendritic cell differentiation and keratinization (Fig. 2).

### LncRNA NCK1-AS1 is specifically up-regulated in cervical cancer and associate with clinical progression

**Fig. 3:**
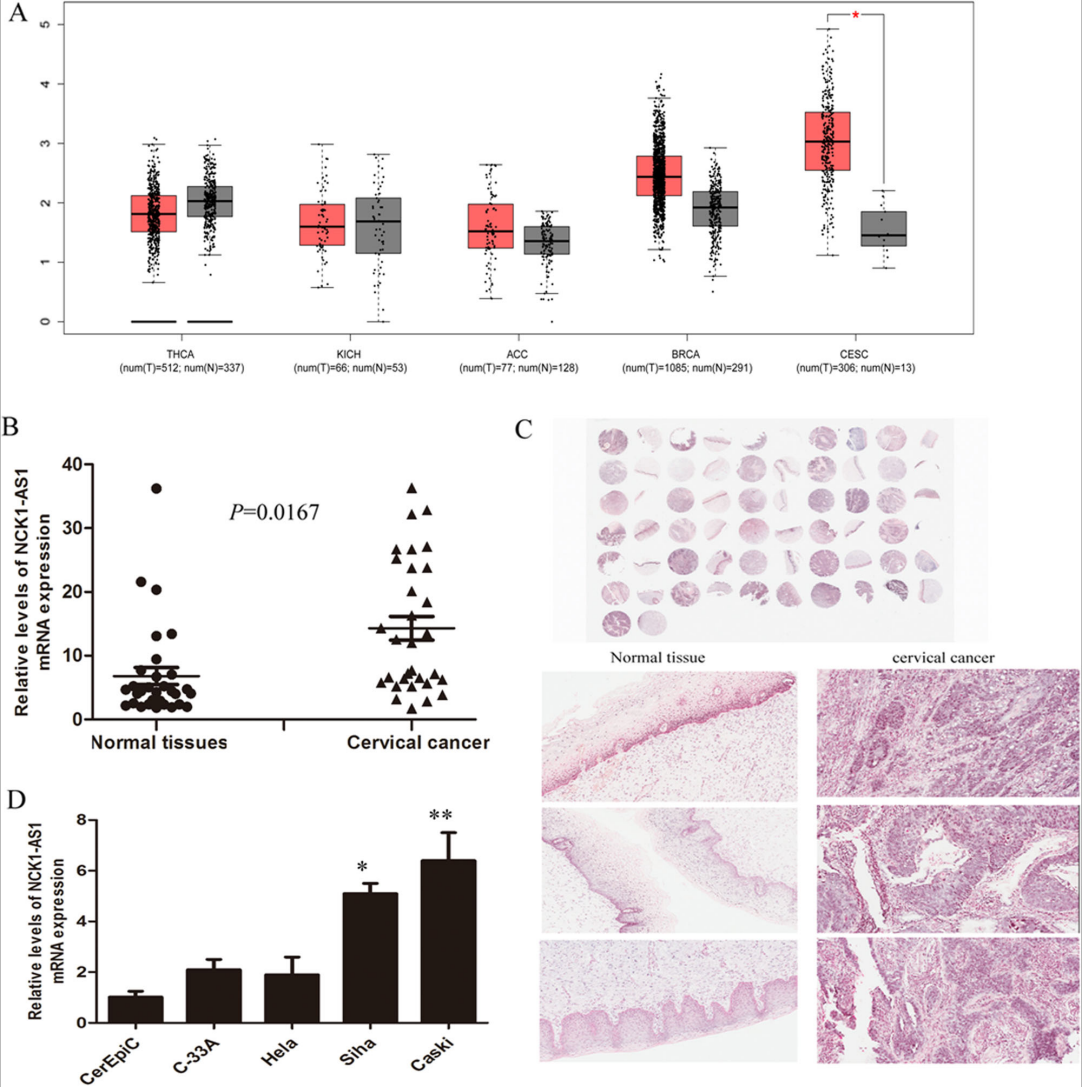
LncRNA NCK1-AS1 is overexpressed in cervical cancer tissues. **a** NCK1-AS 1 expression levels in Thyroid carcinoma (THCA), Kidney Chromophobe (KICH), Adrenocortical carcinoma (ACC), Breast invasive carcinoma (BRCA), and Cervical squamous cell carcinoma and endocervical adenocarcinoma(CESC) using TCGA sequencing data. **b** NCK1-AS1 expression was analyzed by qRT-PCR in CC and adjacent nontumor tissues (*n* = 31). **c** NCK1-AS1 expression in CC tissues and adjacent non-tumor tissues was detected by RNA in situ hybridization. **d** Expression of NCK1-AS1 in cervical cancer cell lines normal human cervical epithelial cells (HCerEpiC), three low metastatic cervical cancer cell lines (HeLa, C33A, and SiHa) and one highly metastatic cell lines (CaSki)

Furthermore, we analyzed the expression of NCK1-AS1 and NCK1 in five cancers (Thyroid carcinoma, Kidney Chromophobe, Adrenocortical carcinoma, Breast invasive carcinoma, Cervical squamous cell carcinoma, and endocervical adenocarcinoma) using TCGA sequencing data sets, showing that NCK1-AS1 is up-regulated in CC tissues (Fig. 3a). However, the expression of NCK1 shows no difference between CC and normal tissue (Supplementary Fig. 1A). The correlation analysis revealed that NCK1 has no correlation with NCK1-AS1 in TCGA CESC Tumor data set (Supplementary Fig. 1B). To validate the expression of NCK1-AS1 in cervical cancer, qRT-PCR and RNA in situ hybridization (RISH) assay was performed to detect the level of NCK1-AS1 in 31 paired CC tissues and adjacent cancer normal tissues. Results confirmed that NCK1-AS1 was highly up-regulated (*P* < 0.01) in 77.4% (24/31) of the CC tissues compared with the normal tissues (Fig. 3b, c). We also examined the expression of NCK1-AS1 in normal human cervical epithelial cells (HCerEpiC), three low metastatic cervical cancer cell lines (HeLa, C33A, and SiHa) and one highly metastatic cell line (CaSki). Higher expression level of NCK1-AS1 was observed in cervical cancer cell lines compared with the human cervical epithelial cells. The highest level of NCK1-AS1 was in the metastatic cancer cell lines CaSki (Fig. 3d).

To investigate the relationship between NCK1-AS1 expression and cervical cancer clinical features, 31 patients were divided into high and low NCK1-AS1 expression level groups based on the median value. Statistical analysis showed no correlations between NCK1-AS1 expression and age, lymph node numbers, tumor size, or clinical stage (*P* > 0.05). Interestingly, the NCK1-AS1 expression level was significantly associated with histological type (*P* < 0.05) and lymph node status (*P* < 0.05) (Table 2).

**Table 2:** Relationship between NCK1-AS1 expression and clinicopathological factors

Characteristic		NCK1-AS	*P*-value
	High expression	Low expression	
Age(year)			0.175
≥50	7	7	
<50	10	7	
Lymph node numbers			0.419
≥15	15	6	
<15	6	1	
Lymph node status			0.0144
N0	18	3	
N1	4	3	
Histological type			<0.0001
malignant	24	7	
normal	5	26	
Tumor size(cm)			0.644
<3	9	2	
≥3	15	3	
Clinical stage			0.823
I	3	1	
II	19	3	
III	4	1	

### Identification of NCK1-AS1 promoter region and transcription factor binding sites

**Fig. 4:**
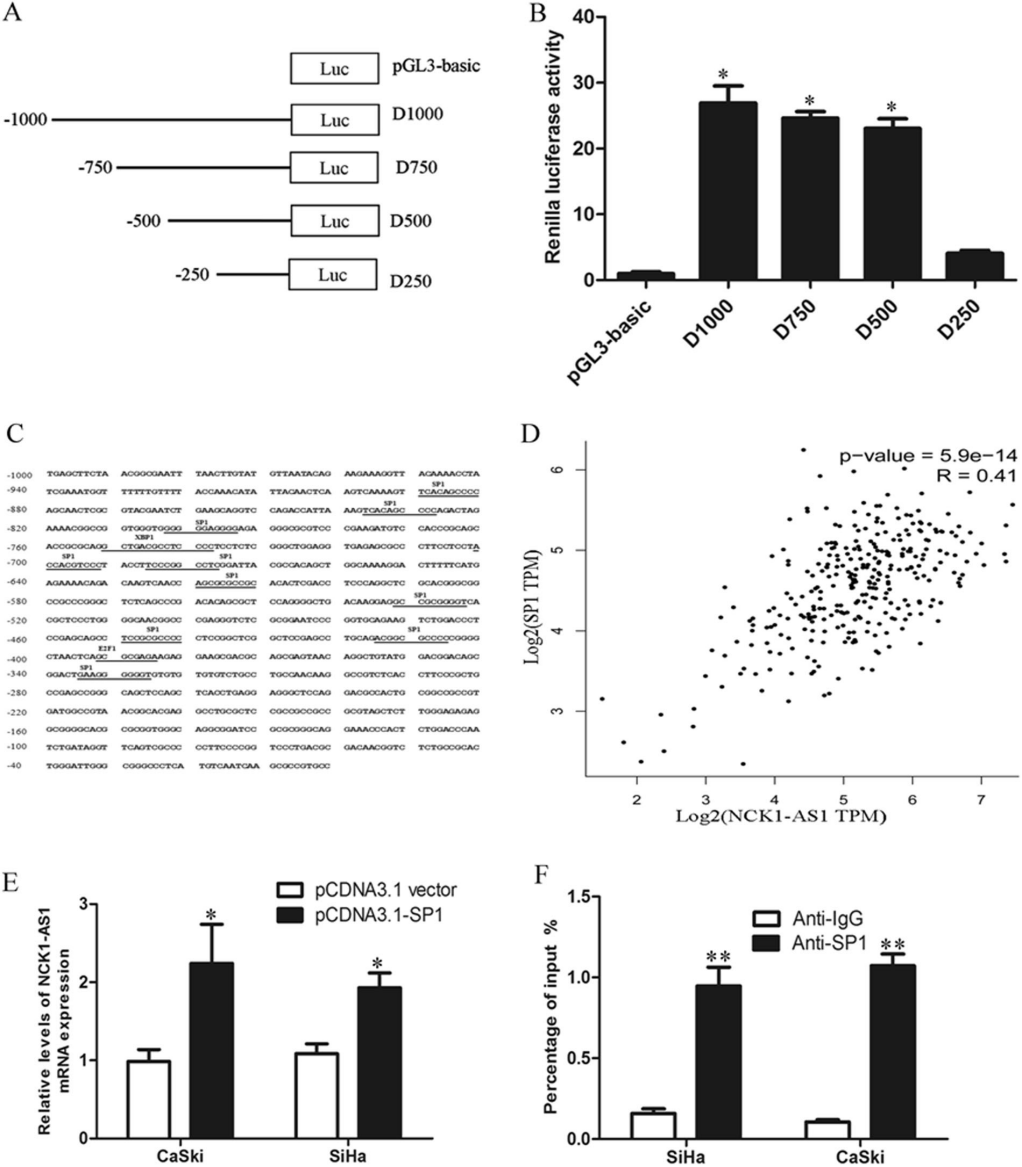
NCK1-AS1 expression was activated by SP1. **a** A schematic diagram of NCK1-AS1 upstream 1000 bp promoter reporter constructs. **b** Dual luciferase reporter assays were used to determine the key promoter activity region of NCK1-AS1. **c** Prediction of SP1 binding sites in the NCK1-AS1 promoter region using JASPAR. **d** The correlation between SP1 and NCK1-AS1 expression was detected by analyzing TCGA data. **e** NCK1-AS1 expression was detected by qRT-PCR in CaSki and SiHa cells transfectd with pCDNA3-vector or pCDNA3-SP1. **f** ChIP assays were performed to detect SP1 directly bound in the NCK1-AS1 promoter region

To identify the core promoter region of the NCK1-AS1 gene, four varieties of luciferase reporter constructs containing the overlapped different fragments of NCK1-AS1 gene 1000 bp region upstream were constructed, as flowing D1000 (−1000–0), D750 (−750–0), D500 (−500–0), and D250 (−250–0) (Fig. 4a). These four luciferase reporter constructs were transfected into SiHa cells and their luciferase activities were measured after 48 h. Dual-Luciferase assay showed that luciferase activities were significant increased in cells transfected with D1000, D750, D500, and D250 compared with the pGL3-basic group. These data demonstrated that a genomic region from−750 to−250 of the NCK1-AS1 gene has a strong promoter activity (Fig. 4b). To further investigate the potential regulators involved in NCK1-AS1 over-expression, potential transcription factor binding sites in the NCK1-AS1 promoter were identified by JASPAR (http://jaspar.genereg.net/). As shown in Fig. 4c, E2F1, XBP1, and SP1 binding sites were found in the promoter region of NCK1-AS1. The correlation between the NCK1-AS1 expression and these transcription factors E2F1, XBP1 and SP1 were performed and correlation analysis revealed that NCK1-AS1 has a significantly positive correlation with SP1 in TCGA CESC Tumor data set (Fig. 4d and Supplementary Fig.1C and 1D). SP1 over-expression promoted NCK1-AS1 expression in SiHa and CaSki cells, while up-regulation of E2F1 or XBP1 had no effect on NCK1-AS1 expression (Fig. 4e and Supplementary Fig.1E). To ask whether SP1 directly transactivates NCK1-AS1 expression, ChIP assay was performed. The result demonstrated that SP1 directly bound to the NCK1-AS1 promoter region (Fig. 4f). Furthermore, luciferase report assays indicated that SP1 binds to the D750 and D500 binding site, but not the D250 site (Supplementary Fig. 1F).

### NCK1-AS1 affects cell proliferation, migration and induces cell cycle progression in vitro

**Fig. 5:**
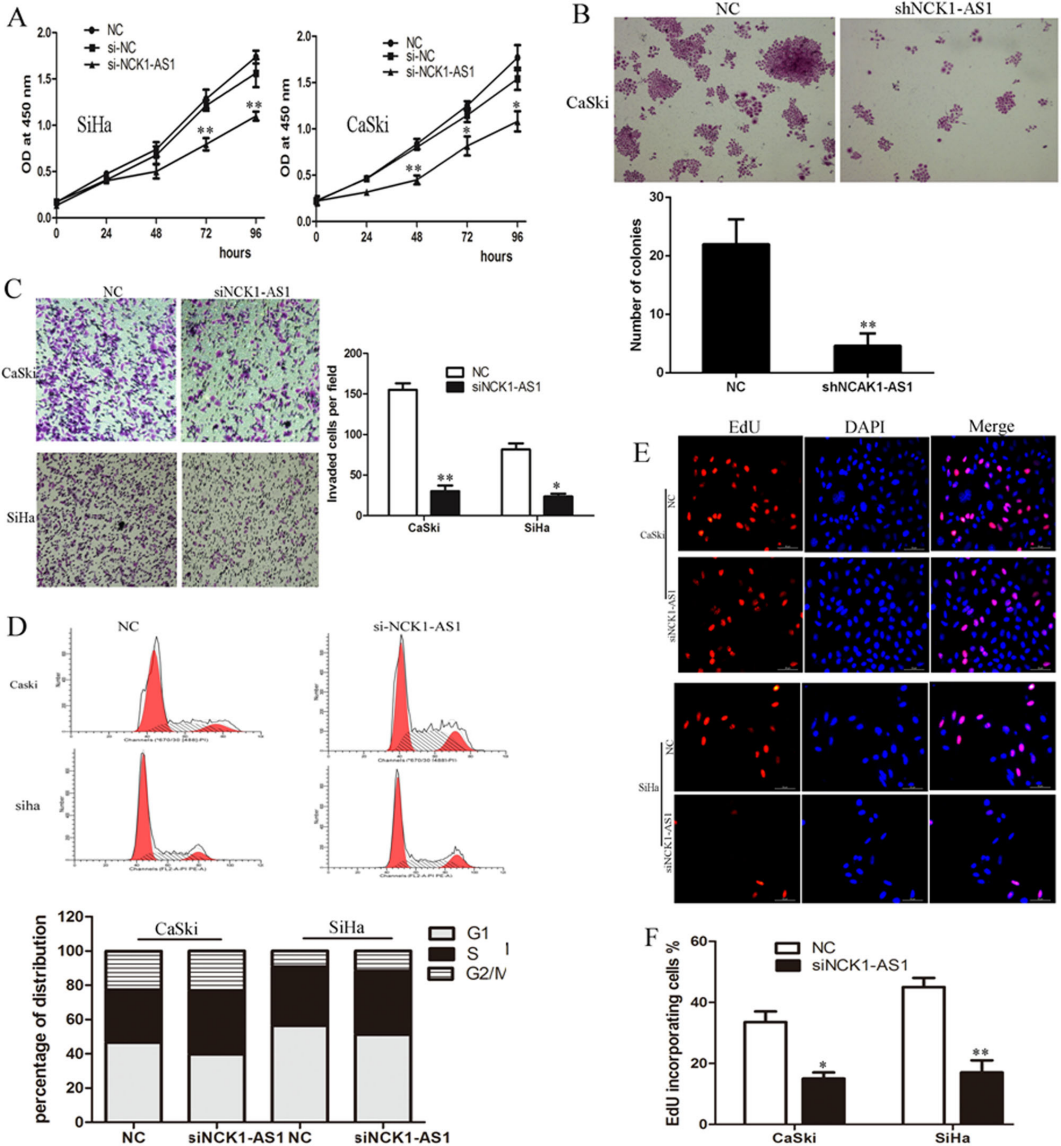
Silencing of NCK1-AS1 promoted CC cell proliferation and invasion and induced cell cycle arrest. **a** CCK-8 assay was performed to detect cell proliferation of control or siNCK1-AS1 transfected CC cells (**b**) colony formation assay was performed to detect cell proliferation of control or stable knockdown NCK1-AS1 CaSki cells. **c** Transwell invasion assays showed that silencing of NCK1-AS1 inhibits CC cell invasion. **d** The effect of knockdown NCK1-AS1 on cell cycle distribution. **e**, **f** micrographs and quantification of EdU-incorporated cells in indicated cells. Error bars represent three independent experiments. *P *<* 0.05, **P *<* 0.01

To validate the function of NCK1-AS1 in regulating cervical cancer cell phenotype, knockdown of NCK1-AS1 in CaSki and SiHa cells that with high NCK1-AS1 expression was carried out via siRNA/shRNA mediated silencing and cell cycle profile and proliferation were subsequently analyzed. qRT-PCR analysis confirmed that the NCK1-AS1 expression level were significantly knocked down in two cell lines, (Supplementary Fig. 2A). qRT-PCR and western blot showed NCK1 expression level did unchanged after silencing of NCK1-AS1 (Supplementary Fig. 2b, c). As shown in Fig. 5a, CCK-8 assays showed that silencing of NCK1-AS1 significantly inhibited CC cell proliferation in vitro. Colony formation assay revealed that stable knockdown of NCK1-AS1 dramatically inhibited anchorage-independent growth abilities in CaSki, as the number and the size of formed colonies of the knockdown NCK1-AS1 cells were far fewer and smaller than those of control cells (Fig. 5b). Transwell assays showed that knockdown of NCK1-AS1 dramatically decreased cell invasion in CaSki and SiHa cells (Fig. 5c). Moreover, flow cytometry (FCM) and EdU immunofluorescent stain were performed to determine if NCK1-AS1 was involved in cell cycle regulation. The percentages of cells were significant increased in G1-phase but decreased in S-phase in CaSki and SiHa cells with NCK1-AS1 knockdown (Fig. 5d). Consistently, EdU stain incorporation assay confirmed this result. Less EdU-positive cells with newly synthesized DNA (15% and 17% respectively) were detected in CaSki and SiHa cells with NCK1-AS1 knockdown compared with that in CaSki and SiHa control cells (38% and 48%, respectively, Fig. 5e, f). These data demonstrated that NCK1-AS1 affected the G1-S transition of cell-cycle progression and inhibited the proliferation, migration and invasion of CC cells.

### Depletion of NCK1-AS1 inhibits CC cell tumorigenesis in vivo

**Fig. 6:**
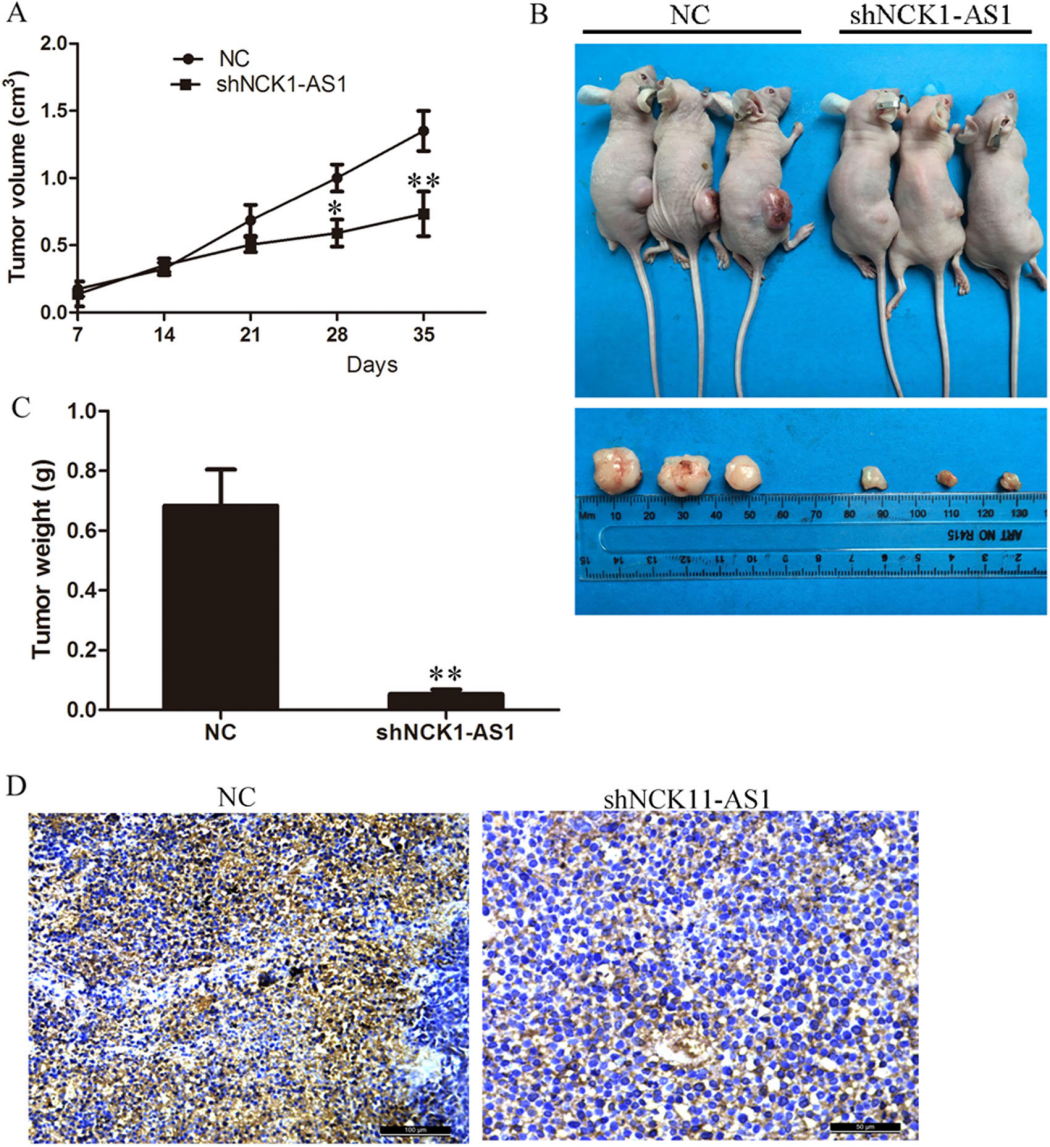
Knockdown of NCK1-AS1 inhibits CaSki cell tumor growth in vivo. **a** the tumor volume growth curves after knockdown NAK1-AS1 cells and control CaSki were injected subcutaneously into the dorsal flanks of nude mice. **b** Representative images of mice bearing tumors from knockdown NAK1-AS1 cells and control CaSki. **c** The tumor weight was measured at the end of the experiment. **d** Ki67 immunostaining of tumor samples from knockdown NAK1-AS1 groups and control CaSki groups. *P *<* 0.05, **P < 0.01

To further validate the role of NCK1-AS1 in the tumorigenesis of cervical cancer, NCK1-AS1 stable knockdown CaSki cells or control cells were injected into nude mice. The result showed knockdown of NCK1-AS1 expression dramatically inhibited the tumor growth in both weight and size in nude mice (Fig. 6a, b). At the end of this experiment, tumor weight of NCK1-AS1 stable knockdown group (0.683 ± 0.121 g) was only 7% of the control group (0.053 ± 0.015 g) (Fig. 6c). Moreover, immunohistochemistry showed that tumor tissues of the NCK1-AS1 knockdown group had fewer Ki67-positive cells than the control group (Fig. 6d).

### Gene expression microarray analysis of NCK1-AS1 knockdown in CaSki cells

To further explore the potential molecular mechanisms of NCK1-AS1 in CC cells, Human Transcriptome Array 2.0 analysis was performed to investigate the differential gene expression profiles between the NCK1-AS1 knockdown group and the control group in CaSki cells. The microarray raw data used in this study have been submitted to National Center for Biotechnology Information (NCBI) Gene Expression Omnibus (GEO) and are accessible through series accession number GSE107171 (http://www.ncbi.nlm.nih.gov/geo/query/acc.cgi?acc=GSE107171)

**Fig. 7:**
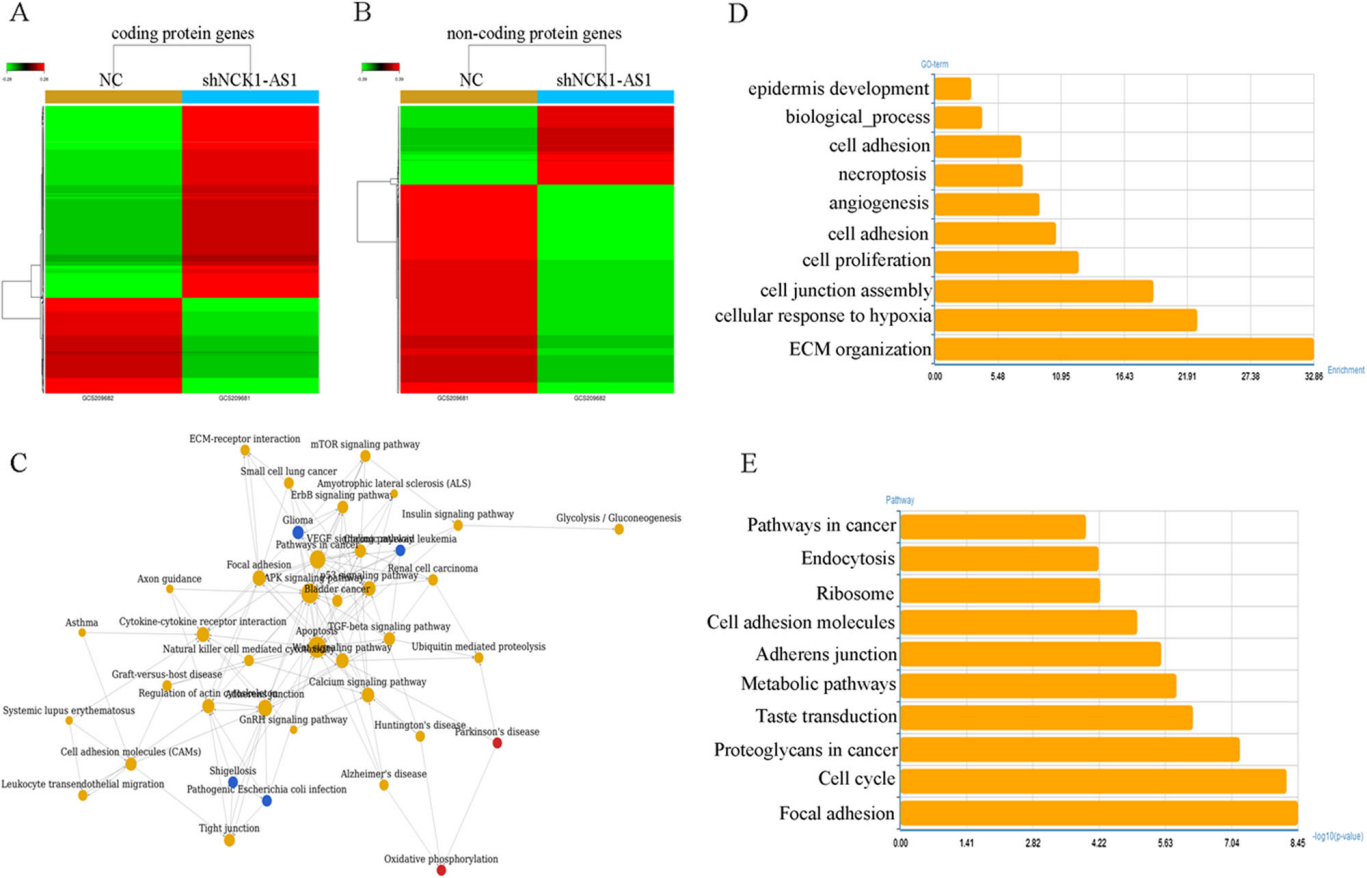
Microarray analysis of transcription profile changes after silencing NCK1-AS1. **a**, **b** Heatmap of altered genes in NCK1-AS1 knockdown CaSki cells compared with control cells. **c** PathwayRelationNetwork of differently expressed 493 coding-genes. **d**, **e** GO and pathway enrichment analysis indicated the differentially expressed 493 genes

By bioinformatics’ analysis, 493 coding-genes and 413 non-coding genes were differentially expressed under the condition of “Q<0.001 and fold change >1.3”. All differentially expressed genes are clustered in Fig. 7a, b. Among the differentially expressed 493 coding protein genes, 165 genes were up-regulated (ratio, >2.0), and 328 genes were down-regulated (ratio, <0.5); while 301 genes were up-regulated (ratio, >2.0), and 112 genes were down-regulated (ratio, <0.5) among differentially expressed 413 non-coding protein genes. The top twenty genes’ expression signatures in knockdown NCK1-AS1 CaSki cells are shown in Supplementary Table 1. PathwayRelationNetwork showed that these coding protein genes are related to the MAPK signaling pathway, cell cycle and Pathways in cancer (Fig. 7c). The Gene Ontology (GO) functional and Kyoto Encyclopedia of Genes and Genomes (KEGG) pathway enrichment analysis indicated the differentially expressed 493 coding protein genes were enriched in extracellular matrix organization, cellular response to hypoxia, cell junction assembly, cell proliferation and epidermis development (Fig. 7d, e).

### NCK1-AS1 functions as ceRNA and sponges of miR-6857 in CC cells

**Fig. 8:**
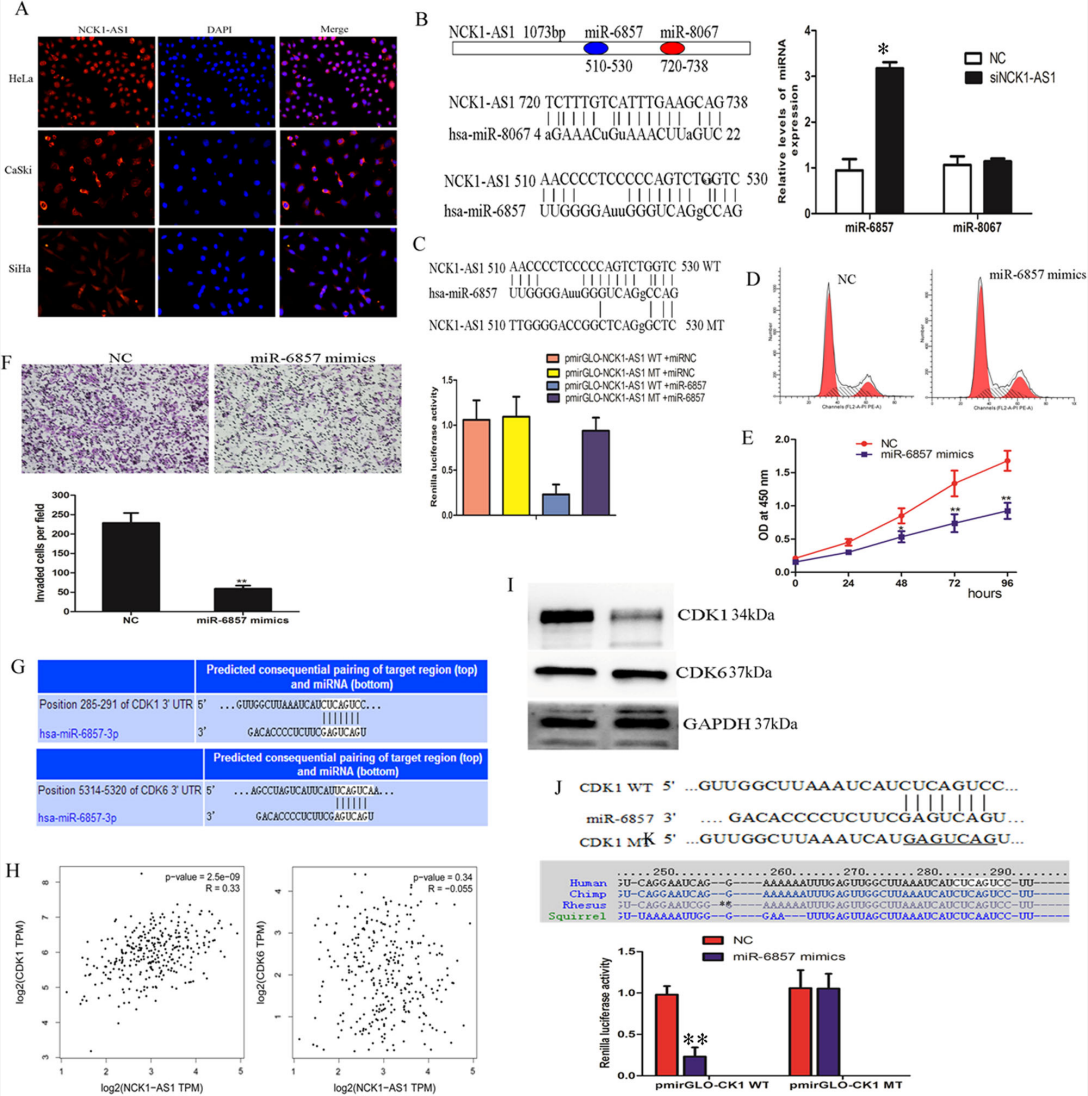
NCK1-AS1 function as a ceRNA for miR-6857. **a** RNA-FISH was used to detect NCK1-AS1 location in CaSki, SiHa and HeLa cells cells. Red, NCK1-AS1; blue, DAPI **b** Left, Schematic outlining the predicted binding sites of miRNAs on NCK1-AS1. Right, qRT-PCR showed that knockdown of NCK1-AS1 dramatically increased the expression level of miR-6857 **c** Luciferase activity in CaSki cells co-transfected with miR-6857 and luciferase reporters containing NCK1-AS1 or mutant transcript. Data are presented as the relative ratio of firefly luciferase activity to renilla luciferase activity. **d**, **e** FCM and CCK8 assays showed that cell cycle and proliferation after over-expression miR-6857 in CaSki cells. **f** Transwell invasion assays showed that cell invasion was suppressed by miR-6857 overexpression in CaSki cells **g** CDK1/6 were predicted downstream targets of miR-6857 **h** The correlation between NCK1-AS1 and CDK1/6 expression was analyzed **i** Western blot analysis of CDK1/6 protein levels in CaSki cells transfected with NC or miR-6857 mimics **j** The complementary sequences or mutant (underlined) binding site of CDK1 and evolutionary conservation of the miR-6857 binding site in CDK1 3-UTR from different mammalian species. *P *<* 0.05, **P < 0.01

Several recent epigenetical studies have suggested that lncRNAs may act as competing endogenous RNAs (ceRNAs) to interact with miRNAs and influence the expression of these miRNAs in cell cytoplasm. Therefore, RNA-fluorescence in situ hybridization (RNA-FISH) was performed to examine its distribution and subcellular fractionation and founded that NCK1-AS1 was distributed in the cytoplasm in CaSki, SiHa and HeLa cells (Fig. 8a). To investigate the underlying mechanism of NCK1-AS1 function, we analyzed the lncRNA NCK1-AS1 sequence and founded miR-6857 and miR-8067 binding sites using the TargetScan prediction algorithm (http://www.targetscan.org/vert_71/) (Fig. 8b). Therefore, we hypothesized that NCK1-AS1 competed to bind with miR-6857 and miR-8067 as a miRNA sponge, and then regulated the expression of downstream targets in CC cells. Correction analysis showed that miR-6857 expression inversely correlated with NCK1-AS1 expressions in the TCGA cervical cancer data sets (Supplementary Fig. 2D). qRT-PCR showed that knockdown of NCK1-AS1 dramatically increased the expression level of miR-6857, while NCK1-AS1 over-expression decreased the expression level of miR-6857, no change of the expression level of miR-8067 (Fig. 8b). For further confirmation, we constructed two dual-luciferase reporters containing: (1) Luc-NCK1-AS1-WT (2) Luc-NCK1-AS1 MT (mutated on the putative miR-6857 sites). As expected, over-expression of miR-6857 reduced the luciferase activities of the WT reporter vector but not empty vector or mutant reporter vector (Fig. 8c). Also, we wished to whether the exogenous over-expression of NCK1-AS1 induces a more malignant phenotype in cervical cancer. Structures of full-length and mutant (mutated on the putative miR-6857 sites) were generated (Supplementary Fig.3A). Hela cells constitutively GFP tagged full-length NCK1-AS1 (LV-NCK1-AS1), or GFP-tagged NCK1-AS1 lacking putative miR-6857 binding sites (LV-NCK1-AS1 MT), (Supplementary Fig.3B). We next assessed the effects of NCK1-AS1 over-expression on invasion and cell proliferation. Results showed that LV-NCK1-AS1 HeLa cells were significantly more invasive than both LV-NCK1-AS1 MT and wild type HeLa cells (Supplementary Fig.3C and D). Over-expression of full-length NCK1-AS1 promoted the anchorage-independent growth of HeLa cells (Supplementary Fig.3E). However, over-expression of miR-6857 counteracted invasion and proliferation in LV-NCK1-AS1 HeLa cells (Supplementary Fig.3F).

To understand the tumor suppressor function of miR-6857, CCK8 and cell cycle assay were performed after miR-6857 control or mimics were transduced into the CaSki cells. As shown in Fig. 8d, miR-6857-overexpressing CaSki cells displayed a significant increase in the percentages of cells in G2/M-phase but decreased proportions in S-phase cells. MiR-6857 over-expression inhibits the propagation of the CC cells as analyzed using CCK8 assay (Fig. 8e). Transwell assays were performed to detect the role of miR-6857 on invasion, and results showed that over-expression of miR-6857 decreased CaSki cell invasion (Fig. 8f)

To address the mechanism underlying the suppressive effect of miR-6857 on the cell cycle of CC cells, we found that cell-cycle promoting genes, including CDK1/6, were examined as downstream targets of miR-6857 using TargetScan, a bioinformatic tool for miRNA target screening (Fig. 8g). Correlation analysis in CC tissues showed that NCK1-AS1 expression is positively associated with CDK1 expression, but not CDK6 (Fig. 8h). CDK1 is a catalytic subunit of the highly conserved protein kinase complex known as M-phase promoting factor (MPF), which is essential for G1/S and G2/M phase transitions of eukaryotic cell cycle. We also found that a putative miR-6857 binding site on the 3-UTR of CDK1 was highly conserved in some species. Western blot assays also showed that knockdown of NCK1-AS1 decreased CDK1 protein levels, which is consistent with miR-6857-induced down-regulation of CDK1 protein (Fig. 8i). Finally, we constructed luciferase reporters containing the putative miR-6857 binding sites, including wild-type (WT) or mutated miR-6857 binding sites. We found that over-expression of miR-6857 reduced the luciferase activities of the WT reporter vector but not mutant reporter vector (Fig. 8j, k).

**Fig. 9:**
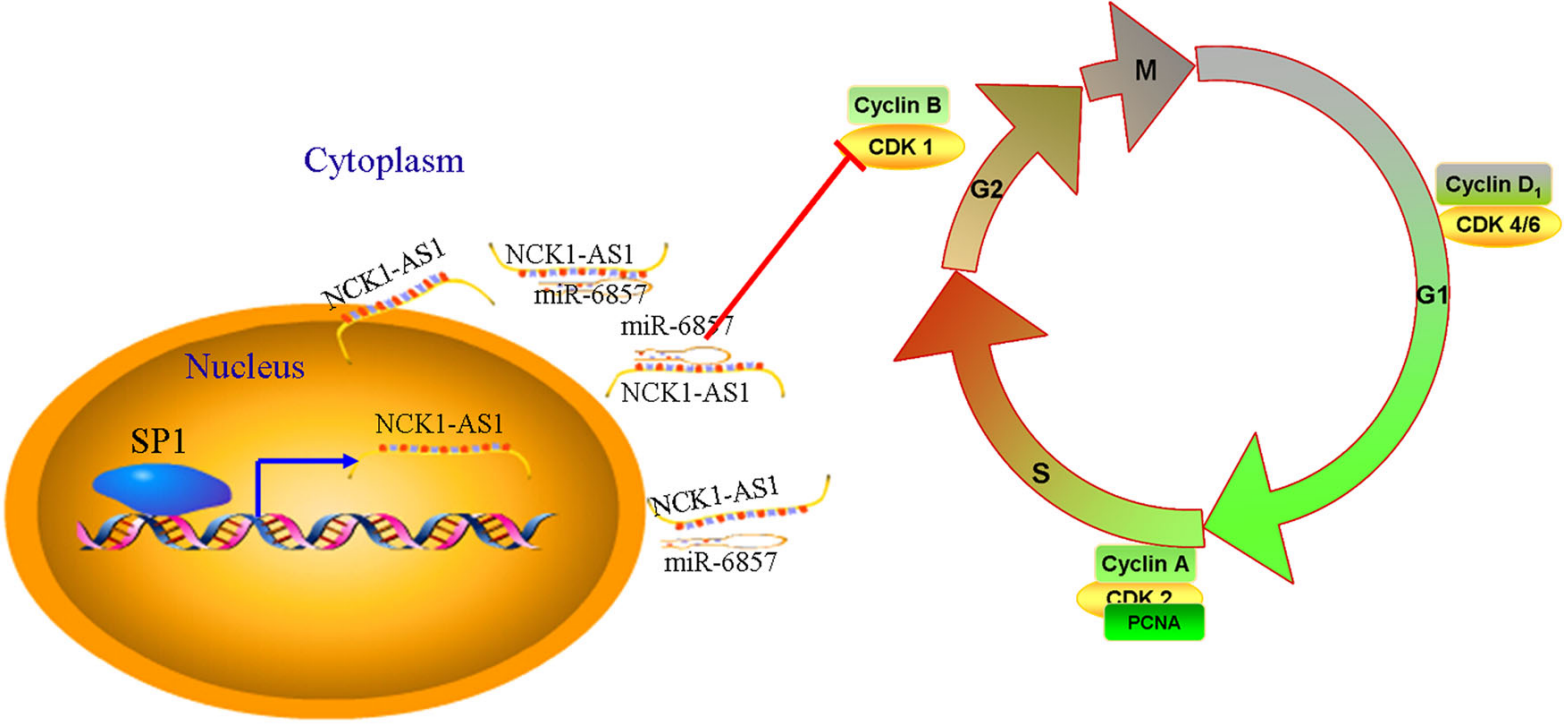
Summary of the mechanism of NCK1-AS1 in CC cells

In conclusion, these data indicate lncRNA NCK1-AS1 downregulated the RNA levels of miR-6857 through directly binding to them thereby derepressing CDK1 expression and imposing an additional level of post-transcriptional regulation (Fig. 9).

## Discussion

Cervical cancer is both the fourth-most common cause of cancer and the fourth-most common cause of death from cancer in women worldwide. In 2012, an estimated 528,000 cases of cervical cancer occurred, with 266,000 deaths. This is about 8% of the total cases and total deaths from cancer. About 70% of cervical cancers occur in developing countries^5^ and in low-income countries, it is the most common cause of cancer death^1^. Although alterations of coding-protein genes in CC development are already a recognized phenomenon, little more than expression patterns of lncRNAs have been established. lncRNAs are a type of non-coding RNAs (ncRNAs) that exceed 200 nucleotides in length. lncRNAs are the relatively abundant component of the mammalian transcriptome and have been implicated in several cellular functions, including the regulation of gene transcription through the recruitment of chromatin-modifying enzymes^21,31,32^. Increasing evidence suggests that these transcripts are frequently and aberrantly expressed in cancers, and some of them have been implicated in diagnosis and prognostication^33,34^.

In the present study, we examined the associations between the lncRNAs expression profile and clinical outcomes of CC patients using the sequencing data from TCGA project and identified a four-lncRNA signature that was significantly associated with the DFS. Based on our established risk-score formula, patients were divided into low-risk and high-risk groups according to the median risk score as cut-off in the training and testing set and patients in the high-risk group had significantly shorter median DFS than those in the low-risk group. We found that the lncRNA NCK1-AS1 has specific over-expression in cervical cancer tissue but not in other cancers. qRT-PCR and RISH result confirmed the NCK1-AS1 expression pattern in CC clinical samples and NCK1-AS1 expression was significantly associated with histological type and lymph node status. However, the transcription factors involved in NCK1-AS1 disorder expression in cervical cancer are not well known. In this study, our data demonstrated that SP1 directly bound to the NCK1-AS1 promoter region and activated NCK1-AS1 expression.

Previous studies demonstrated that lncRNAs play key roles in regulation of the malignant phenotypes of cancer cells. To further investigate the functions of lncRNA NCK1-AS1 in CC cells, we performed the loss-of-function evaluation in two CC cell lines (CaSki and SiHa) with higher NCK1-AS1 expression. Our findings showed that knockdown of NCK1-AS1 inhibited CC cell proliferation and invasion, and induced cell cycle arrest in vitro. We also showed depletion of NCK1-AS1 inhibits CC cell tumorigenesis in vivo. RNA-fluorescence in situ hybridization (RNA-FISH) showed NCK1-AS1 was to be distributed in the cytoplasm. Human Transcriptome Array 2.0 analysis showed 493 coding-genes and non-coding genes were differentially expressed between NCK1-AS1 knockdown group and control group in CaSki cells. Moreover, we found NCK1-AS1 functions as a ceRNA for miR-6857 in the cytoplasm, and binding with miR-6857 releases its inhibition of CDK1/6 mRNA, resulting in elevated CDK1/6 protein levels.

Taken together, our study revealed that the GC-associated lncRNA NCK1-AS1 is an oncogenic lncRNA which promotes tumorigenesis through function as ceRNA for specific miRNAs. These data support the idea that crosstalk involving lncRNA NCK1-AS1/miR-6857/CDK1 plays key roles in cervical cancer progression and potentially work as a therapy target.

## Materials and Methods

### RNA sequence processing and lncRNA profile mining

The level3 sequencing of cervical squamous cell carcinoma and endocervical adenocarcinoma and corresponding clinical data were downloaded from The Cancer Genome Atlas (TCGA) (https://cancergenome.nih.gov/). GATExplorer was used to process microarrays for gene expressions of lncRNAs. Gene expression data for other cancers (Kidney Chromophobe, Thyroid carcinoma, Adrenocortical carcinoma, Breast invasive carcinoma) were downloaded from the TCGA data set.

### LncRNA in situ Hybridization

Surgical specimens of this study included tissues from 31 cervical squamous cell carcinoma and thire adjacent non-cancerous cervical squamous epithelium tissues were obtained from 31 patients who underwent potentially curative surgery in the First Affiliated Hospital of Chongqing Medical University from July 2013 to June 2015. The expression of NCK1-AS1 was examined by *RISH* in 31 pairs of paraffin-embedded tissues, which were cut into 4 μm thick, and 2 mm diameter, sections to construct tissue microarrays (TMA). Briefly, the TMA were digested with proteinase K, and hybridized with double digoxin-labeled LNA^TM^-modified NCK1-AS1 probe (Exiqon, Vedbaek, Denmark) overnight at 55 °C, then incubated overnight at 4U with an anti-Digoxigenin-AP, Fab fragments (Roche, Basel, Switzerland 200:l). The cells nuclei were stained with NBT/BCIP (Roche, Basel, Switzerland) in the dark. Specific NCK1-AS1 ISH signals were identified as brown, punctate dots, and expression level was scored as Image-Pro Plus 6.0 software.

### Cells culture

Human cervical epithelial cells (CerEpiC) from ScienCell Research Laboratories were cultured in Cervical Epithelial Cell Growth Supplement (CerEpiCGS, Cat #7062), a complete medium designed for optimal growth of normal cervical epithelial cells in vitro. Four human cervical cancer cell lines ((HeLa, C33A and SiHa and CaSki) were obtained from Chinese Type Culture Collection, Chinese Academy of Sciences, were cultured in DMEM medium (Hyclone, Massachusetts, USA), and were supplemented with 10% fetal bovine serum (Hyclone, Massachusetts, USA), 100 U/ml penicillin sodium under an incubator with an atmosphere of 5% CO_2_/95% air at 37 °C.

### RNA interference

The sequences of the siRNAs used to suppress NCK1-AS1 expression were sense 5′−GAAUGUCAUCCCAGCCGAATT−3′, antisense 5′−UUCGGCUGGGAUGACAUUCTT−3′. The control siRNA sequence that targeted green fluorescent protein (GFP) were sense 5′−UUCUCCGAACGUGUCACGUTT−3′, antisense5′−ACGUGACACGUUCGGAGAATT−3′. The sequences of the short hairpin RNA used to suppress NCK1-AS1 expression were sense 5′−GATCCGAATGTCATCCCAGCCGAATTCAAGAGATTCGGCTGGGATGACATTCTTTTTTG−3′, antisense 5′−AATTCAAAAAAGAATGTCATCCCAGCCGAATCTCTTGAATTCGGCTGGGATGACATTCG−3′. siRNAs were obtained from GenaPharma (Shanghai, China). siRNA and Lentviral3-GFP-shRNA specfilly targeting NCK1-AS1 and negative control were designed and synthesized by Genepharma (Shanghai, China). Transfection of siRNA (60 μM) was carried out using Lipofectamine® RNAiMAX Reagent (Invitrogen) according to the manufacturer’s instructions. Lent virus3-GFP-shRNA was used to infect CaSki cells. After infection 24 h, CaSki cells were cultured in complete media containing puromycin (3 μg/ml) to generate a stable knockdown NCK1-AS1 cell line.

### Quantitative real-time PCR

The following primer sequences were used→to detect→each gene: 5′−GGAGCGAGATCCCTCCAAAAT−3′ and 5′−GGCTGTTGTCATACTTCTCATGG−3′ for GAPDH, 5′−TTCCCATTTCTCCCAGGTCC−3′ and 5′−TGGTTACTTTGAGCCTGCCT−3′ for NCK1-AS1, 5′- AGAGTGGTGGAAATGCAGGA−3′ and 5′−TTGATGCCTGGTG ACTTTGC−3′ for NCK1, 5′−TGGCAGCAGTACCAATGGC−3′ and 5′−CCAGGTAGTCCTGTCAGAACTT−3′ for SP1, 5′−CCAGGAGTTACTTCTATGCCTGA−3′ and 5′−TTCATCCAGGGGAGGTACAAC−3′ for CDK2, 5′−AAACTACAGGTCAAGTGGTAGCC−3′ and 5′−TCCTGCATAAGCACATCCTGA−3′ for CDK1. miRNA reverse-transcription and the expression levels was performed by miRNA First Strand cDNA Synthesis Tailing Reaction Kit (B532451,Sangon Biotech, Shanghai, China) according to the manufacturer’s instruction. Universal miRNA qRT-PCR Primer 5′−AACGAGACGACGACAGAC−3′, 5′−GCAAATTCGTGAAGCGTTCCATA−3′ for RNU6, 5′−TGGGGATTGGGTCAGGCC−3′ for has-miR-6857, 5′−TAATAG CTCAGAATGTCAGTTCTG−3′ for hsa-miR-7705, 5′−ATGGGGACAGGGATC AGCAT−3′ for hsa-miR-6810. The reactions were performed using the SYBR Premix Ex TaqTM II (TIi RNaseH Plus,TAKARA), and the CFX Connect Real Time System (BIO-RAD) was used for analysis. Relative expression was normalized to the endogenous control GAPDH or RNU6 using the 2^−ΔΔCt^ method.

### Cell proliferation and invasion assays in vitro

Cell proliferation ability was determined by Cell counting Kit-8 (CCK8) (Dojindo Laboratories,Japan) after siRNA transient transfection for 24, 48 and 72 h. Colony formation assays were performed to monitor the cloning capability of stable knockdown NCK1-AS1 CC cells cloning capability. Two weeks later, colonies were fixed with methanol and stained by 0.25% crystal violet staining solution. For the invasion assay, cells were serum-starved overnight and 2 × 10^4^ cells were seeded in a Matrigel-coated chamber and cultured for 48 h. The invaded or migrated cells were fixed with 70% methanol and stained 0.25% crystal violet staining solution. Cells invading to the lower surface of filters were counted in five randomly selected fields. All experiments were carried out in triplicate.

### Tumor growth assay in vivo

The animal study protocol was approved by the Animal Experimentation Ethics Committee of Chongqing Medical University. Six female Balb/c nude mice (aged four weeks) were provided by Beijing Laboratory Animal Research Center (Beijing, China) and housed in a pathogen-free animal facility (Laboratory Animal Center of Chongqing medical university). Briefly, six nude mice were randomly assigned to the control or experimental group (three mice per group). 1 × 10^6^ shCtrl and shNCK1-AS1 CaSki cells were suspended in 0.14 ml of phosphate buffer solution and subcutaneously injected into the femoral area of nude mice. The tumor was measured with calipers and the volume was calculated using the formula: (π/6)*x*^3^, where *x* = the largest diameter. Thirty-five days after tumor inoculation, the mice were sacrificed and the tumors were extracted to determine tumor weight. Data are presented as the mean ± SD.

### Fluorescence in situ hybridization analysis

Cy3 labeled FISH probes were designed and synthesized by Genepharma (Shanghai, China). Cells were grown on glass coverslips, fixed with ice-cold 4% paraformaldehyde for 20 min, and blocked with Pre-hybridization Buffer for 30 min. Cells were incubated with 25 μM NCK1-AS1 FISH probe in hybridization buffer in Hybridization Buffer in the dark at 37 °C overnight, washed three times with 2 × SSC, incubated with DAPI for detection for 15 min according Fluorescent In Situ Hybridization Kit manufacturer (Ribo, Guangzhou, China), and then analyzed using a confocal fluorescence microscope (FV1000-D, Olympus, Tokyo, Japan).

### Luciferase reporter assay

The SP1 binding motif in the promoter region of NCK1-AS1 was identified by JASPAR (http://jaspar.genereg.net/). The full-length and the different fragment sequences were synthesized and then cloned into the pGL3-basic vector (Promega, Madison). Cells were seeded into 24-well plates and then cotransfected with 100 ng of pmirGLO-CDK1-WT, pmirGLO-CDK1-mut, and 200 nmol/L of miR-6857 mimics or control by using Lipofectamine 2000. Luciferase assays were performed 48 h after transfection using the dual-luciferase reporter assay system (Promega). Luciferase activities were assessed using the Dual Luciferase Assay Kit (Promega), according to the manufacturer’s instructions.

### Statistical analysis

IBM SPSS statistics 23 was used for statistical analysis of cell invasion, colony formation, and tumor formation in the mice and conducted using the Student *t* test. The association between NCK1-AS1 expression and tumor stage was determined by Chi-square test. *P* ≤ 0.05 was considered as statistically significant.

## Electronic supplementary material

Supplementary
