# Supplementary material for: LncRNA NCK1-AS1 promotes proliferation and induces cell cycle progression by crosstalk NCK1-AS1/miR-6857/CDK1 pathway
Source: Cell Death Dis. 2018 Feb 7;9(2):198. doi: 10.1038/s41419-017-0249-3 (PMC5833418; doi:10.1038/s41419-017-0249-3)
Supplement: Supplementary file 1 — Supplementary [file 41419_2017_249_MOESM1_ESM.doc]

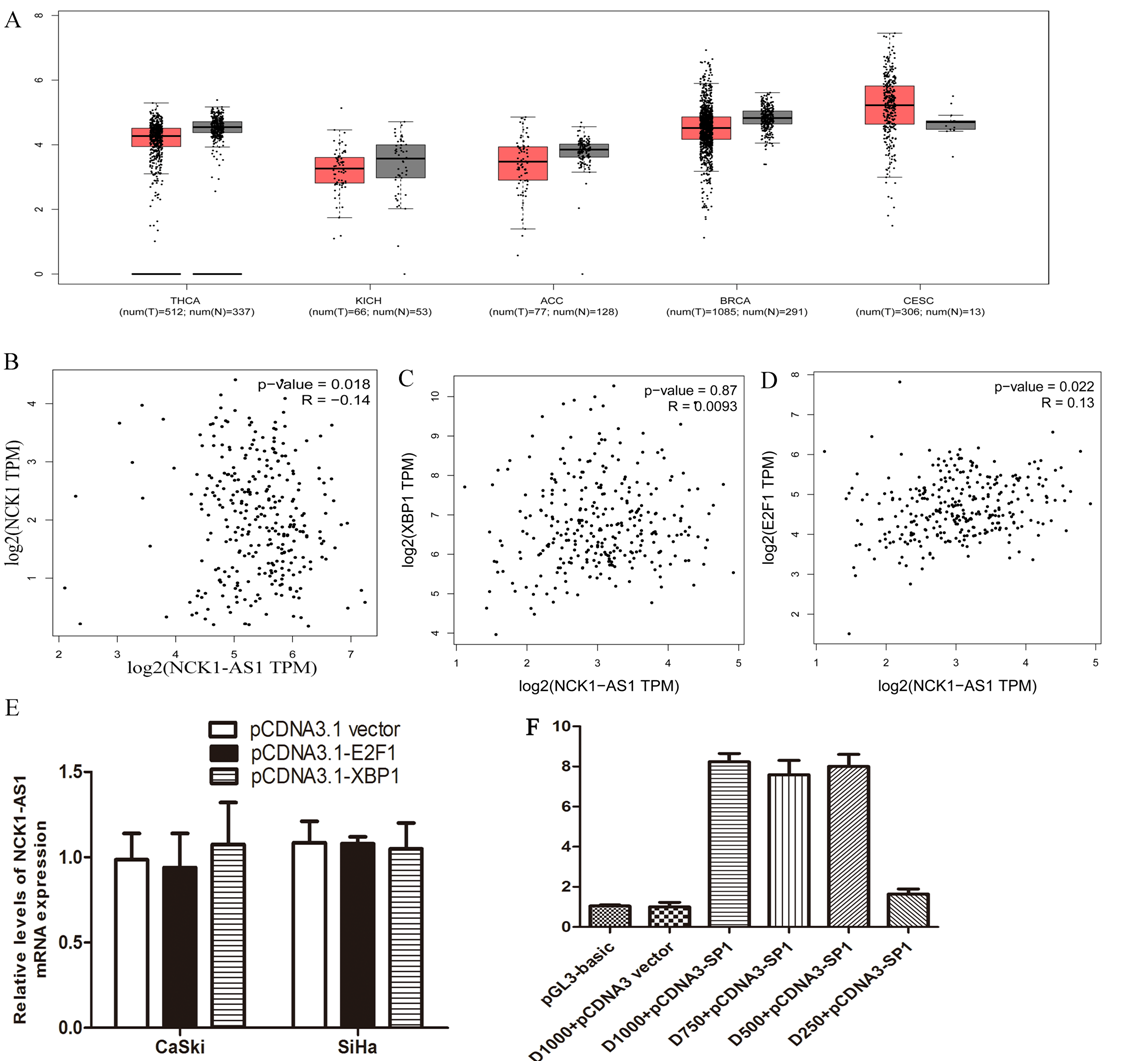


Supplementary Fig.1 (A) Analyses of NCK1-AS 1 expression levels in Thyroid carcinoma (THCA), Kidney Chromophobe(KICH), Adrenocortical carcinoma(ACC), Breast invasive carcinoma (BRCA) and Cervical squamous cell carcinoma and endocervical adenocarcinoma(CESC) using TCGA sequencing data. (B) The correlation between the NCK1 and NCK1-AS1 expression were performed using TCGA CESC Tumor dataset. (C, D) The correlation between E2F1, XBP1 and NCK1-AS1 expression was detected by analyzing TCGA data (E) NCK1-AS1 expression was detected by qRT-PCR in CaSki and SiHa cells transfectd with pCDNA3-vector or pCDNA3-E2F1 or pCDNA3-XBP1 (F) luciferase report assays indicated that SP1 binds to the D750 and D500 binding site, but not the D250 sites


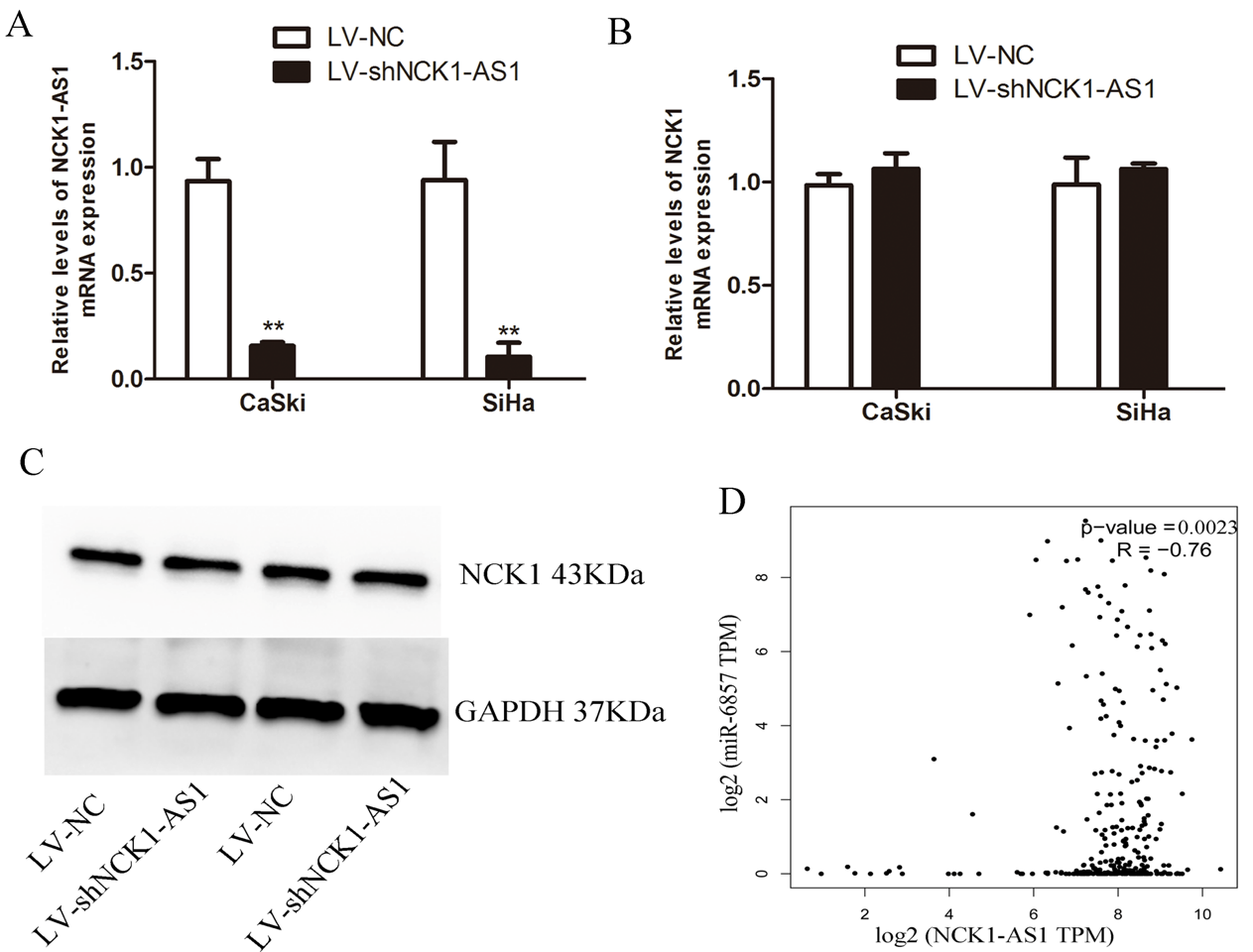


Supplementary Fig.2 (A) NCK1-AS1 expression levels was significantly knockdown in two cell lines (B, C) qRT-PCR and western blot was detected NCK1 expression no changed after silenced NCK1-AS1 (D) The correlation between the NCK1-AS1 and miR-6857 expression were performed using TCGA CESC Tumor dataset.


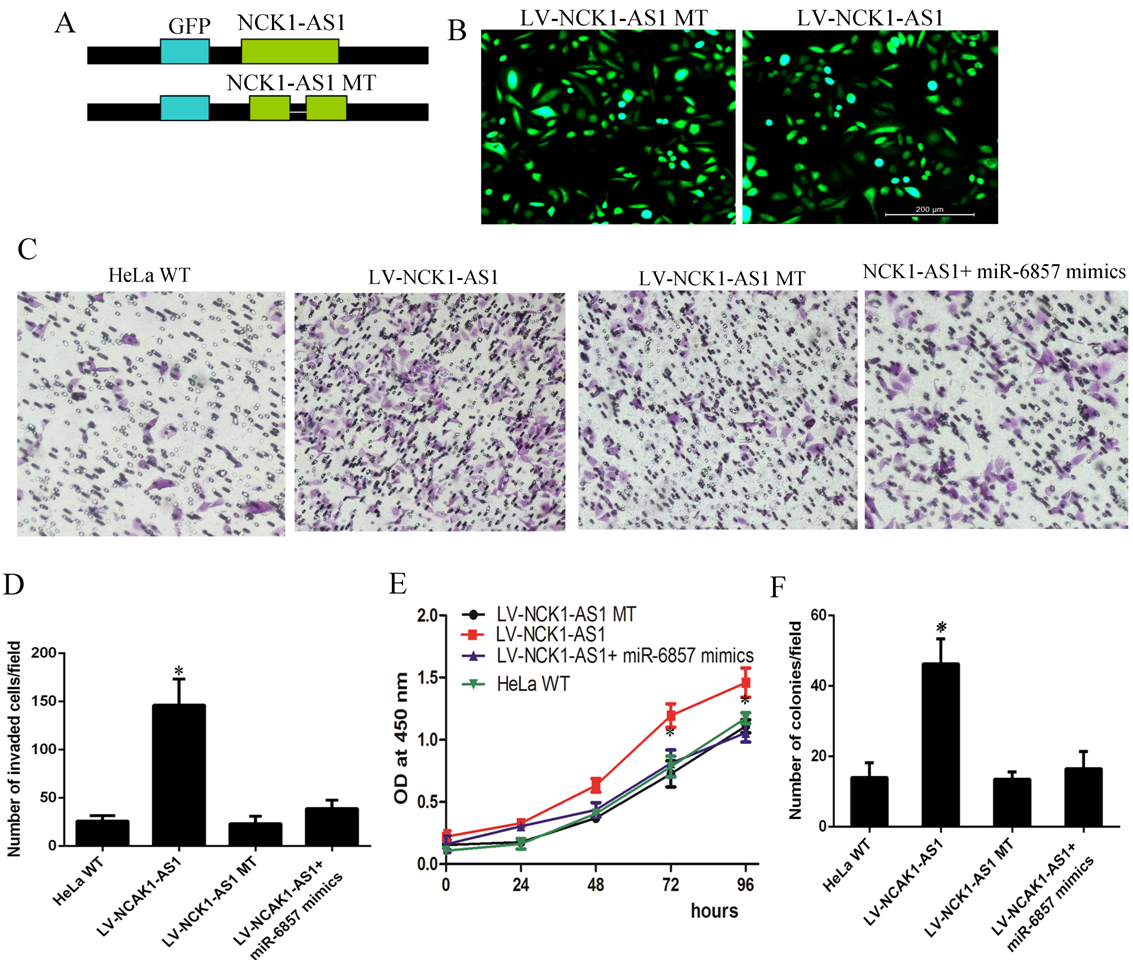


Supplementary Fig.3 Exogenous expression of NCK1-AS1 in HeLa cells promotes cell invasion and proliferation. (A) Structures of full-length and mutantNCK1-AS1. (B)HeLa cells constitutively expressing GFP-tagged full length ALX1 (FL) or GFP-tagged NCK1-AS1 deleted of mutated on the putative miR-6857 sites were generated by lentiviral infection. (C, D) the invasive properties of HeLa cells were evaluated using an in vitro invasion assay. The graph shows the average number of invaded cells per field. Three independent experiments were carried out, and the data are represented as the mean±SD (**P* < 0.05). (E, F) HeLa cells were detected to proliferation assay. The average number of colonies per field is indicated in the graph. Three independent experiments were carried out, and the data are represented as the mean±SD (**P* < 0.05).

Supplementary table1 Top twenty genes expression signature in knocckdown NCK1-AS1 CaSki cells

| Probe Set ID | Gene Symbol | Fold Change | Biotype |
| --- | --- | --- | --- |
| TC19000266.hg.1 | CASP14 | -2.49061 | coding |
| TC16000559.hg.1 | CDH1 | -2.39939 | coding |
| TC01006383.hg.1 | CDK1 | -2.04675 | coding |
| TC01002338.hg.1 | HTR1D | -2.0229 | coding |
| TC15000971.hg.1 | ALDH1A3 | -1.94589 | coding |
| TC09000614.hg.1 | PTGS1 | -1.94411 | coding |
| TC12001239.hg.1 | CDK4 | -1.94105 | coding |
| TC11002346.hg.1 | MPZL2 | -1.89489 | coding |
| TC01003771.hg.1 | MUC1 | -1.87939 | coding |
| TC12001243.hg.1 | TAS2R30 | -1.86969 | coding |
| TC01001563.hg.1 | XPR1 | -1.8589 | coding |
| TC10002963.hg.1 | ANXA8L1 | -1.83627 | coding |
| TC02002492.hg.1 | GALNT3 | -1.83407 | coding |
| TC08001659.hg.1 | NDRG1 | -1.82462 | coding |
| TC10000314.hg.1 | ANXA8L1 | -1.81925 | coding |
| TC12000822.hg.1 | TCP11L2 | -1.81588 | coding |
| TC03001641.hg.1 | | 1.789026 | coding |
| TC12001718.hg.1 | PTPRB | -1.73374 | coding |
